# IMPROVING LONG-TERM CARE THROUGH GERIATRICS EDUCATION: A DEEP DIVE INTO GWEP AND GACA INITIATIVES

**DOI:** 10.1093/geroni/igad104.3426

**Published:** 2023-12-21

**Authors:** Gina Tucker-Roghi, Rajean Moone, Christopher Hernandez, Hannah Moerk, Linda Edelman, Gail Towsley

**Affiliations:** Dominican University of California, San Rafael, California, United States; University of Minnesota, Minneapolis, Minnesota, United States; University of Utah, Salt Lake City, Utah, United States; University of Utah, Salt Lake City, Utah, United States; University of Utah, Salt Lake City, Utah, United States; University of Utah, Salt Lake City, Utah, United States

## Abstract

Formal geriatrics education remains limited for many of the numerous health care professionals working with older adults. A well-prepared long-term services and supports (LTSS) workforce is imperative to care for older adults with multiple chronic conditions, especially dementia. This study highlights the role of Geriatric Workforce Enhancement Programs (GWEPs) and Geriatrics Academic Career Awards (GACAs) in addressing this need. An online survey, conducted 5/2023-6/2023, captured data from GWEPs (n=18) and GACAs (n=11) about training offered towards age- and dementia-friendly care in LTSS settings. Results revealed that 13 GWEPS or GACAs provided 36 learning activities that addressed the first recommendation, “Delivering comprehensive, person-centered, equitable care that ensures residents’ health, quality of life and safety” of the National Imperative to Improve Nursing Home Quality (NASEM). Twelve GWEPS/GACAs provided 42 learning activities addressing the second NASEM report recommendation, “ensuring a well-prepared, empowered, and appropriately compensated workforce.” GWEPS/GACAs are also aiding LTSS settings in achieving Age-Friendly Health Systems Designations: one each supported level 1 or 2 designation, two supported achieving both levels. Additionally, four GWEP/GACAs are working with LTSS entities toward level 1 status, one toward level 2, and one toward achieving both levels. Our findings underscore the pivotal role of GWEPS/GACAs in enhancing geriatrics education, addressing challenges in LTSS, and the pressing need for continued support. Recommendations emphasize strengthening partnerships between LTSS settings and GWEPs/GACAs to bridge educational gaps. Implications indicate GWEPS and GACAs are playing a crucial role in helping LTSS settings achieve age-friendly standards and align with the NASEM report’s vision.

